# Genomics, Proteomics, and Metabolomics Approaches to Improve Abiotic Stress Tolerance in Tomato Plant

**DOI:** 10.3390/ijms24033025

**Published:** 2023-02-03

**Authors:** Bindu Naik, Vijay Kumar, Sheikh Rizwanuddin, Mansi Chauhan, Megha Choudhary, Arun Kumar Gupta, Pankaj Kumar, Vivek Kumar, Per Erik Joakim Saris, Muzamil Ahmad Rather, Shuvam Bhuyan, Panchi Rani Neog, Sadhna Mishra, Sarvesh Rustagi

**Affiliations:** 1 Department of Food Science and Technology, Graphic Era (Deemed to Be) University, Bell Road, Clement Town, Dehradun 248002, Uttarakhand, India; 2 Himalayan School of Biosciences, Swami Rama Himalayan University, Swami Rama Nagar, Jolly Grant, Dehradun 248014, Uttarakhand, India; 3 Department of Life Sciences, Graphic Era (Deemed to Be) University, Bell Road, Clement Town, Dehradun 248002, Uttarakhand, India; 4 Department of Microbiology, Dolphin (PG) Institute of Biomedical and Natural Sciences, Dehradun 248007, Uttarakhand, India; 5 Department of Microbiology, Faculty of Agriculture and Forestry, University of Helsinki, FI-00014 Helsinki, Finland; 6 Department of Molecular Biology and Biotechnology, Tezpur University, Tezpur 784028, Assam, India; 7 Faculty of Agricultural Sciences, GLA University, Mathura 281406, Uttar Pradesh, India; 8 Department of Food Technology, Uttaranchal University, Dehradun 248007, Uttarakhand, India

**Keywords:** abiotic stress, climate variability, metabolic reactions, phytohormones, defense feedback, transcriptional changes, metabolomics, microbial interaction

## Abstract

To explore changes in proteins and metabolites under stress circumstances, genomics, proteomics, and metabolomics methods are used. In-depth research over the previous ten years has gradually revealed the fundamental processes of plants’ responses to environmental stress. Abiotic stresses, which include temperature extremes, water scarcity, and metal toxicity brought on by human activity and urbanization, are a major cause for concern, since they can result in unsustainable warming trends and drastically lower crop yields. Furthermore, there is an emerging reliance on agrochemicals. Stress is responsible for physiological transformations such as the formation of reactive oxygen, stomatal opening and closure, cytosolic calcium ion concentrations, metabolite profiles and their dynamic changes, expression of stress-responsive genes, activation of potassium channels, etc. Research regarding abiotic stresses is lacking because defense feedbacks to abiotic factors necessitate regulating the changes that activate multiple genes and pathways that are not properly explored. It is clear from the involvement of these genes that plant stress response and adaptation are complicated processes. Targeting the multigenicity of plant abiotic stress responses caused by genomic sequences, transcripts, protein organization and interactions, stress-specific and cellular transcriptome collections, and mutant screens can be the first step in an integrative approach. Therefore, in this review, we focused on the genomes, proteomics, and metabolomics of tomatoes under abiotic stress.

## 1. Introduction

*Solanum lycopersicum* L. (Solanaceae), generally known as tomato, is one of the most significant fruits that are nutritionally classified as a vegetable. It contains carotenoids (lycopene and carotene), phenolic compounds (flavonoids), vitamins (ascorbic acid, -tocopherol, vitamin A) [1], glycoalkaloids (tomatine), and phytosterols (-sitosterol, campesterol, and stigmasterol) [2,3]. Fernandes et al. (2021) [4] stated the high percentage of polyunsaturated and monounsaturated fatty acids such as palmitic and arachidic acid, oleic and linolenic acids, and stearic acids residing in tomatoes. Furthermore, the tomato has a significant amount of lycopene pigment, which protects both the tomato and the consumer from UV radiation. It also functions as an antioxidant that boosts immune responses and lowers cholesterol levels, resulting in a healthy heart. Lycopene can also be used to treat periodontitis and gingivitis. Because of the presence of zeaxanthin and lutein, tomatoes are also known to protect the eyes and lower the risk of macular degeneration.

Being such an essential cure and a trove of benefits; tomatoes are in huge demand. They are most likely to get affected by unfavorable growth conditions and phytopathogens. To induce its yield, it is quite important to study the effect of various stress parameters such as low temperatures, drought, and salinity, and naturally integrate their resistance mechanisms within the crop. Abiotic stress is studied as the leading cause of crop loss globally, lowering average yields for most agricultural plants by more than 50% [5] and prohibiting plants from achieving their full genetic potential.

Introduction to advanced technology has resulted in the appearance of various multidimensional omics disciplines, including genomics, transcriptomics, proteomics, metabolomics, etc., which deal with the molecular components of cellular life. The advancements in genomic studies have aided in the discovery of various gene families and mechanisms that affect tolerance to abiotic stressors and hence boost yield. These genes may be part of protein kinases such as mitogen-activated or calcium-reliant, and they further activate transcription factors (TFs) and cis-acting elements that regulate stress-response patterns [6]. Moreover, combined research on plant–environment interactions provides a comprehensive perspective of molecules at the cellular, tissue, or organism level [7]. The integration of several omics techniques that provide significant biological information is being tackled through system biology, a relatively emerging discipline of life science. Systems biology in combination with virtual experiments provides visualization and comprehension of how plants cope with abiotic stress [8]. In addition, designing climate-smart cultivars for high and steady production in unfavorable climatic situations necessitates the integration of multidisciplinary expertise. In this regard, the current study presents detailed information on recent breakthroughs in proteomics, as well as strategies for the effective investigation of accessible resources in tomatoes dealing with abiotic stress resistance.

Finding a research question based on the genomes, proteomics, and metabolomics of tomatoes was conducted for the review. Google Scholar, Science Direct, Scopus, Web of Science, Springer Link, and Wiley were the databases used in this investigation. Proteomics, genomics, metabolomics, tomato omics, abiotic stress, genes for abiotic stress, proteins for abiotic stress, and metabolites for abiotic stress were the keywords utilized in the study. Using a set of quality criteria and exclusion criteria (published before 2017, duplicate papers already retrieved, papers not in English), the relevant studies were filtered after being chosen. In the end, the retrieved data were synthesized in response to the research questions after all pertinent data from the chosen studies were extracted and presented in this review.

## 2. Major Abiotic Stress in Tomato

**Figure 1:**
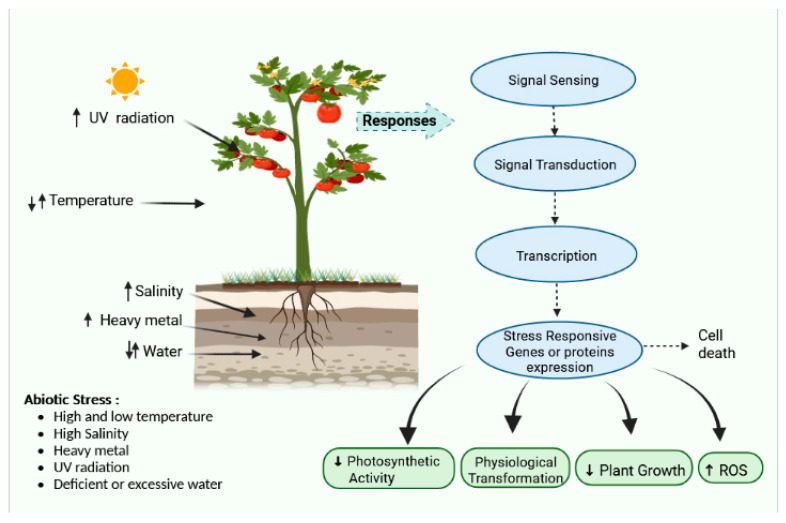
Effect of various stresses and their responses and pathways.

Abiotic stress is a broad term that encompasses a variety of conditions such as temperature variations, osmotic pressure, drought, salinity, water logging, bruising, ozone, toxic compounds, excessive light, and UV radiation [9]. These are serious issues and cause a cascade of morphological, physiological, molecular, and biochemical changes, resulting in cellular damage and inhibition of general metabolism. Such environmental stressors include various factors that behave as ecological barriers that tremor cellular stability. They possess a negative impact on plant development and agricultural output [10]. Owing to the complexity of stress and its origin, finding particular abiotic stress is unusual; therefore, in responses to various stressors, a large number of stress-responsive components and pathways are prevalent (Figure 1).

Temperature is an important abiotic factor that regulates cellular homeostasis. Heat increases water evaporation from the soil together with a greater leaf transpiration rate, resulting in water stress under field circumstances; it also increases the kinetics of biomolecules, which may result in protein misfolding [11], whereas cold reduces biomolecule and enzyme kinetics, resulting in lower cell membrane fluidity. Snow develops ice crystals in the soil, freezing the water residing within.

Water availability is the most significant abiotic element that has influenced and continues to influence plant evolution, and by 2050, more than half of all arable lands may be severely affected [12]. Drought reduces soil water balance, hampering osmotic adjustment, which is characterized by a reduction in the osmolarity of cytosol caused by the accumulation of various hydrophilic proteins (LEA proteins) and osmolytes. Drought causes stomatal closure in leaves, resulting in an imbalance between electron transport mechanisms and carbon absorption, leading to increased thermal energy dissipation and photoinhibition [13]. Anaerobiosis caused by flooding stress causes fermentation in plant roots. The cytoplasm of cells becomes more acidic during fermentation due to an increased concentration of organic acids, which inhibits the functioning of various enzymes. Induced salinity results in the buildup of numerous osmolytes and ions such as Na^+^, which may become toxic when accumulated in large amounts.

Heavy metal soil pollution is a global issue that is continuously becoming worse due to human activity, geochemical rock weathering, and other environmental factors including volcanic activity, acid rain, etc. Metal-contaminated soil is constantly being exposed to plants. The harmful effects include the adhesion of heavy metal ions to the sulfhydryl groups of proteins, deactivation of enzymes, removal of vital cations from specific binding sites, and generation of ROS, which causes oxidative damage to lipids, proteins, and nucleic acids [14]. In addition to limiting agricultural yields, heavy metal absorption poses a serious risk to both flora and fauna [15].

## 3. Physiological Transformation Due to Abiotic Stress in Tomato

**Figure 2:**
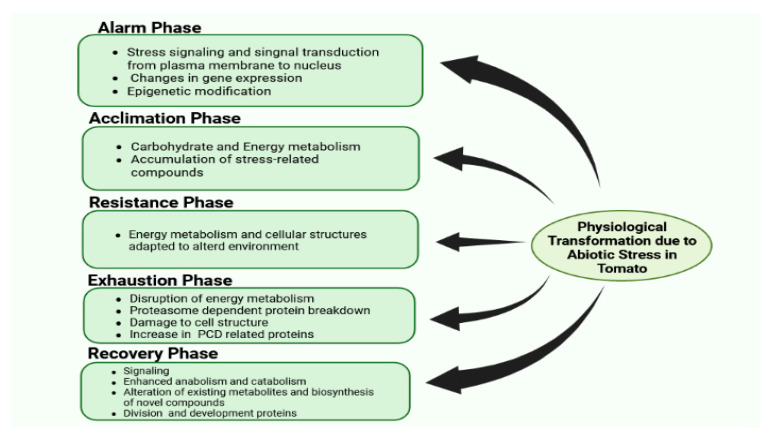
Physiological transformation due to abiotic stress in tomato.

There are certain phases of stress in plants, such as alarming, acclimation, resistance, exhaustion, and recovery. The differing proteome response exhibited in every phase is summarized in Figure 2. Despite this, other physiological, cytosolic, and molecular responses can also be observed, which are elaborated on in upcoming sections.

According to various reports, proteins are involved in stress signaling (NDPK, involved in G-protein signaling and phosphate transfers), ROS scavenging (thioredoxin, glyoxalase I), detoxification, and chaperones (DnaK and HSP20); moreover, proteins are responsible for cell wall biosynthesis such as enzymes of the phenylpropanoid biosynthesis pathway [16]. Additionally, several cytoplasmic proteins, such as pentose phosphate pathway proteins and glucose metabolism, were found in the cell membrane. It is suggested that they might have a role in the manufacture of sugars as a component of osmotic alteration during dehydration stress or in ROS scavenging owing to NADPH generation.

ROS (singlet oxygen, superoxide, hydroxyl radicals, and hydrogen peroxide) are inherent byproducts released continuously during metabolic pathways; their formation and degradation are often reported to increase under environmental stresses [17], although ROS are also crucial communicating molecules [18] for numerous processes taking place in specific cell organelles. In drought-exposed sugar beets, cellular drying is also related to increased ROS production, leading to the activation of various ROS scavenging enzymes, including multiple thioredoxin (Trx) isoforms [19]. Accumulation of ROS causes oxidative stress, which destroys plant biomolecules and cellular components affecting the growth potential of plants [20].

Due to the closing of stomata, CO_2_ fixation is reduced, which causes a decrease in NADP^+^ regeneration via the Calvin cycle, and when this gets combined with altered photosystem activity as well as photosynthetic transport potential, it results in greater electron permeability to O_2_ [21,22]. Similar proteins such as GRP-like proteins-2 and APX (ascorbate peroxidase), as well as GPX (glutathione peroxidase), were discovered in the cell wall and leaves of tomatoes as abiotic stress-associated proteins. They are suggested to function as an integrating factor for the accumulation of cell wall elements and defense against oxidative stress respectively [23].

Ferritin- and osmotin-like proteins, which make up 13% of all known proteins, are crucial for detoxification. While ferritin stores iron in a soluble, nontoxic form that is readily accessible, ferrous iron is absorbed in the ferrous form and stored as ferric hydroxides when oxidized. According to reports, plants accumulate osmotin or proteins that resemble osmotin in response to a variety of stressors [24]. Small heat shock proteins (HSP) found in mitochondria make up 7% of all known proteins. The interruption of regular protein synthesis and the persistent production of HSPs are two characteristics of the heat shock response, which entails the temporal alteration of metabolic activity in the cell. The ability of plant mitochondria to withstand heat is greatly influenced by MT-sHSPs [23]. The upregulation of these proteins’ expression shows that they are crucial for avoiding the aggregation of denatured proteins and promoting protein refolding in response to stress [25].

**Figure 3:**
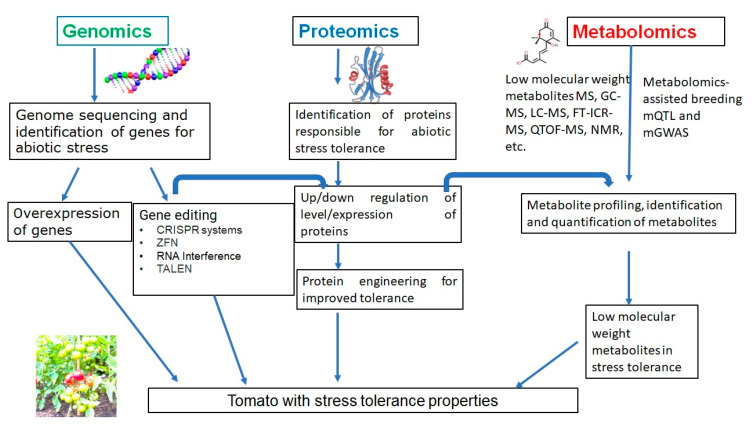
The framework of study techniques for the studies of genomics, proteomics, and metabolomics for tomato.

Li et al. (2019) [26] developed slnpr1 mutants utilizing SlNPR1 from the variety of tomato ‘Ailsa Craig’ via the CRISPR/Cas9 system. The observation indicated various effects such as reduced drought tolerance, greater electrolytic leakage, increased stomatal aperture, hydrogen peroxide (H_2_O_2_) levels, higher malondialdehyde (MDA), and reduced antioxidant enzyme activity. The framework of study techniques and characterization in each section for the studies of genomics, proteomics, and metabolomics for tomato is given in Figure 3.

## 4. Genomics of Abiotic Stress

Solanaceous crop genomics is in an exciting stage of development after the sequencing of the potato and tomato genomes was recently completed. Tomato, a native of South America, has become one of the most widely utilized vegetable crops after spreading over the globe. It exhibits intriguing developmental traits, such as compound leaves, succulent fruits, short generation time, and easy diploid genetics. Together, these factors make the tomato a superb species for both basic and applied plant study [27]. Before the tomato genome sequencing research began, several genetic and genomic materials were available. To build a genetic map of tomatoes, scientists used morphological and isozyme markers [28]. They then determined which 12 linkage groups corresponded to the cytologically accessible chromosomes, which resulted in the building of an RFLP (restriction fragment length polymorphism) linkage map [29]. Therefore, breeders were able to locate quantitative trait loci (QTLs) as a result of the complete molecular linkage map, which helped them comprehend the genetic basis of many quantitative traits (Tomato Genome Consortium, 2012). On the website of the Solanaceae Genomics Network, there is a plethora of information about the genetic and genomic resources for tomatoes.

Recent developments in the genomics field have augmented the effective development of novel varieties. The ability to characterize genetic variation in the germplasm pool for almost any crop species has considerably improved, owing to molecular markers [30]. They are based on the polymorphism observed in any DNA sample, and molecular markers have numerous benefits compared to biochemical or morphological markers. Included in these benefits are simple assays, repeatability, ease of use, high availability, stability regardless of external or environmental conditions, and representation across entire genomes [31]. Numerous applications in genetics, molecular biology, genomics, and plant breeding—particularly tomatoes—have made extensive use of DNA markers. Numerous omics branches dealing primarily with the molecular elements of cellular biology have been developed recently because of technological advancements [32]. The genomics approach offers a comprehensive perspective for the structural–functional investigation of genes and the discovery of genetic variants, which can be employed to overcome abiotic stress in tomatoes.

### 4.1. Genome Sequencing of Tomato

Diverse abiotic stresses in open-field cultivation negatively influence tomato crop production, resulting in poor yield and fruit quality, thus fetching a lower price and not fulfilling the market requirement. To overcome the issues of abiotic stress (especially temperature and flood) in open fields, farmers tend to cultivate tomato crops in greenhouses [33]. In addition to being more expensive, greenhouse farming also results in a rapid buildup of NO_3_, PO_4_, and salt in the soil, which eventually causes the degradation of soil and polluting of the surface and groundwater. Therefore, increasing tomato cultivars’ ability to withstand abiotic stress is economically more advantageous and sustainable [34]. Abiotic stress conditions brought on by harsh water and temperature regimes, an imbalance in the nutritional content of the soil substrate, and elemental toxicity, along with high salinity, are the main issues limiting tomato development. In agricultural settings where many stressors frequently coexist, the abiotic pressures grow increasingly complex. It is vital to create resilient, high-yielding cultivars with improved tolerance to a series of abiotic stresses to meet the world’s food needs [35].

There have been various attempts to address certain stress aspects under controlled conditions, but this approach is not always workable because the plant response varies in the field when numerous factors and stresses are present at once [29,36]. Over the past ten years, conventional breeding has significantly improved a wide range of traits, including biotic and abiotic stress tolerance, yield components, and quality-related variables. However, conventional kinds are susceptible to numerous stresses at diverse locations. Given the genetic complexity and environmental interconnections, using more intensive and multidisciplinary methodologies provides a superior approach to improving stress tolerance in current crops [32]. The genomics of solanaceous crops is in a motivating stage of development after the sequencing of the potato and tomato genomes was recently completed.

### 4.2. Identification and Functional Validation of Genes Associated with Abiotic Stress Tolerance

One of the various strategies available for improving contemporary tomato plants is genetic engineering. Functional genomics has recently undergone technological advancements that have made it possible to identify the many gene families and processes that affect how plants respond to abiotic challenges, and consequently increase yield [27]. Genetic modification (GM) technology, in contrast to conventional selective breeding, enables faster and more effective acquisition of tomato plants resistant to abiotic challenges, increasing food production [27].

Yang et al. (2015) [37] conveyed that both the *SlSnRK22.1* gene intricated in the regulation of abscisic acid signaling and the *SlSnRK2.2* gene involved in osmotic stress signal transduction overexpressed and increased sensitivity to osmotic stress. Yu et al. (2016) [38] studied the low-temperature stress in tomatoes and observed that the *SlMPK7* gene regulated ROS homeostasis through the initiation of cellular antioxidant systems and also modulated the transcription of stress-associated genes, resulting in improved tolerance to chilling. Liu et al. [39] studied the overexpression of *ShDHN*/*Shabrochaites* genes and observed the accumulation of protein with chaperone-like and detergent properties, resulting in enhanced tolerance to cold, drought, and salinity. Li et al. [40] stated that *SpWRKY1* is a transcriptional factor, transcriptional regulation stress-related gene, and its overexpression resulted in enhanced abiotic tolerance. Shah et al. [41] reported that the *AtDREB1A* gene overexpressed under the Lip9 promoter and resulted in the expression of a transcriptional factor, transcriptional regulation stress-related gene, resulting in plant cold resistance. According to Meng et al. [42], overexpression of the *LeAN2* gene led to the upregulation of several structural genes in the anthocyanin biosynthesis pathway, leading to anthocyanin accumulation, which improved heat resistance. The *AtGRX* gene is responsible for maintaining cellular redox homeostasis and is responsible for conferring chilling tolerance to tomato plants [43]. Similarly, Hu et al. [44] reported about the *MdSOS2L1* gene, which is involved in signal transduction proteins and influences ion-driving transport mechanisms, and overexpression of the gene resulted in improved salt tolerance. Gong et al. [45] studied about *SlSAM1* gene in tomatoes, which catalyzes the ATP and L-methionine conversion into S-adenosylmethionine, which is involved in polyamines and ethylene biosynthesis, resulting in plant alkali tolerance. Using small interfering RNA technology, Metwali et al. [46] improved tomato fruit quality under heat stress by silencing the *vis-1* gene.

Researchers from all over the world have used a variety of techniques to produce tomato crops with different levels of abiotic stress tolerance [47]. GRAS transcription factors (TFs) have been shown by Habib et al. (2021) [48] to play an array of roles in biological processes. Abiotic stressors such as drought and salt stress were used to characterize how a tomato *SlGRAS10* gene functions. Dwarf plants possessing shorter internode lengths, smaller leaves, and increased flavonoid accumulation were created when *SlGRAS10* was downregulated via RNA interference (RNAi). These plants also showed heat tolerance. Abscisic acid (ABA), which is connected to members of the abscisic acid-responsive element binding factor (ABF) and abscisic acid-responsive element binding protein (AREB) subfamily of basic leucine zipper (*bZIP*) transcription factors, has been reported to play a significant role in how plants respond to abiotic stresses. The cultivated tomato contains the *SlAREB1* and *SlAREB2* components. Salinity and drought tolerance were promoted in the tissues of the leaves and roots by the overexpression of *SlAREB1* and *SlAREB2* [49]. The authors discovered genes that were upregulated in *SlAREB1*-overexpressing lines that encode proteins related to transcription regulation, oxidative stress, lipid transfer proteins (LTPs), and late embryogenesis abundant protein, showing that these genes might be participating in abiotic stress and could be reactive to pathogenic microbes. As per the structure of the gene, the composition of the motif, and phylogenetic investigation, Wang et al. [28] recognized 66 potential G2-like genes in tomatoes and categorized them into five groups (I to V). All 12 chromosomes had an unequal distribution of the G2-like genes. There were four tandemly duplicated *SlGlk* genes and nine pairs of tandemly duplicated gene segments. The *SlGlks* promoter regions contain a variety of CREs that are connected to hormones and stress, according to the cisregulatory elements (*CREs*) analysis. *SlGlks* were expressed in response to various abiotic stressors according to RNA-seq.

Gene expression, namely *SlDWARF*, *SlCPD*, and *BIN2*, was considerably elevated during drought stress in *SLB3*-silenced seedlings; however, *TCH4*-related gene expression was downregulated, according to Wang et al. [50]. These findings established that silencing the SLB3 gene decreased tomato plants’ ability to withstand drought and had an effect on the BR signaling transduction, which may be likely to blame for the difference in tomato plants’ ability to withstand drought. For biotic and abiotic stress responses. Bvindi et al. [51] examined the functions of tomato histone H3 lysine methyltransferases Set Domain Group 33 (*SDG33*) and *SDG34*. The *H3K36* and *H3K4* methylations, as well as the gene expression involved in a variety of processes and reactions to biotic and abiotic stimuli, were altered in the *SDG33* and *SDG34* mutants. The double mutant demonstrated better tolerance, consistent with separate and additional functions, whereas single mutants were still resistant to drought. Mutants enhanced recovery and survival when the drought ended and maintained a higher water status during it. Notably, decreased trimethylation in *H3K4* and *H3K36* and the production of negative regulators in stressed plants aid in the mutants’ ability to withstand stress. The tomato *PDI* gene family was thoroughly analyzed for the first time at the genome-wide level by Wai et al. [52], who discovered nineteen *PDI* genes in tomatoes, which were distributed unequally across eight tomato chromosomes out of twelve, with segmental duplications found for three paralogous gene pairs. The majority of the *PDI* genes showed variable expression across various fruit organs and developmental phases, according to expression profiling research. Additionally, the majority of the *PDI* genes were strongly activated by high temperature, salinity, and abscisic acid treatments, but only a small number of the genes were stimulated by freezing and stresses, including nutrition and water deficits. Dominate expression of the *PDI* gene family, *SlPDI1-1*, *SlPDI1-3*, *SlPDI1-4*, *SlPDI2-1*, *SlPDI4-1*, and *SlPDI5-1* in response to ABA application and abiotic stresses suggested that they have a role in managing tomato abiotic stresses.

### 4.3. Genomic Approaches to Combat Abiotic Stress

**Figure 4:**
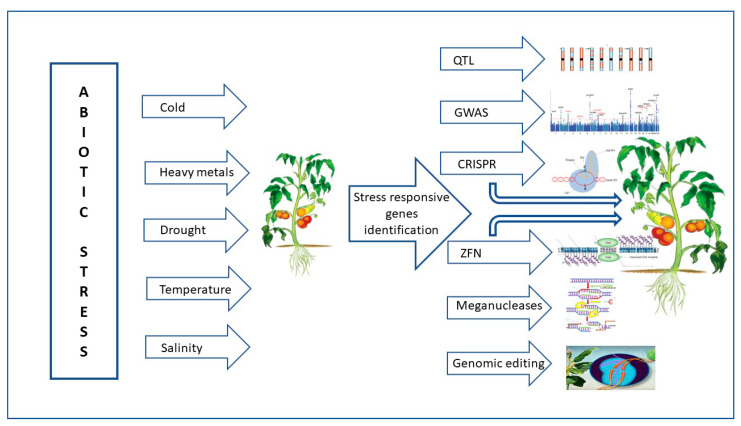
Schematic illustration of the usage of genomic approaches for making abiotic stress-tolerant tomato plants. The abiotic-stressed tomato plant shows restricted growth, while the plant with gene editing exhibits vigorous growth under abiotic stresses. QTL—quantitative trait loci; GWAS—genome-wide association studies; CRISPR—clustered regularly interspaced short palindromic repeats; ZFN—zinc-finger nucleases.

Through a variety of mechanisms, including transcription, translation, regulation of calcium, phytohormone, sugar, and lipid signaling, as well as primary and secondary metabolism, plants respond to high temperatures and maintain life [32]. Numerous abiotic stimuli, such as heat stress during bud development, which results in stigma (style) exertion in tomato flowers, have a significant impact on the position and maturity of the male (anther cone) and female (style) organs (Krishna et al., 2019) [37]. Genetic diversity exists in heat stress resistance. Under heat stress, plants display a variety of physiological reactions, including leaf abscission and senescence, growth retardation of the shoots and roots, and fruit destruction. As a result, plant productivity is significantly reduced [53,54]. Plant development and agricultural productivity are harmed by unfavorable environmental variables such as salt stress, drought, and severe temperatures. GRAS genes are members of the family of plant-specific transcription factors (TFs), which are known to play a variety of functions in plant growth and development. According to several studies, the GRAS protein family is essential for plant growth and development in response to abiotic stressors [55]. The tomato *SlGRAS10* gene’s functional characterization under abiotic conditions such as salt stress and drought was shown by Habib et al. [48]. Dwarf plants with shorter internodes and smaller leaves were created when *SlGRAS10* was downregulated via RNA interference (RNAi). Additionally, compared to wild-type plants, *SlGRAS10-RNAi* plants were more resilient to abiotic stimuli such as salt, dehydration, and abscisic acid. By increasing the tomato plant’s osmotic potential, flavonoid biosynthesis, and ROS scavenging system, the researchers demonstrated the significant role of *SlGRAS10* as a stress-tolerant transcription factor in a particular form of abiotic stress tolerance. A schematic illustration of the usage of genomic approaches for making abiotic stress-tolerant tomato plants is given in Figure 4.

Gisbert et al. [56] demonstrated that tomato plants with the *HAL1* gene, which comes from the yeast *Saccharomyces cerevisiae*, had increased tolerance to salinity. The ability of transgenic tomato lines to retain K^+^ under salt stress was shown to be higher than that of control plants when intracellular cation ratios (K^+^ to Na^+^) were considered. Therefore, by lowering long-term shoot Na^+^ retention, overexpression of the yeast gene *HAL5* in tomatoes increases their ability to withstand salt stress. Regardless of the level of salt stress, this was the result of decreased Na+ transfer from roots to shoots. According to Kumari et al. [57], tomato plants overexpressing *AtNHX1* had higher salt resistance. The researchers suggested that *AtNHX1* was in charge of promoting active K+ absorption at the tonoplast and K^+^ dispersion inside cells.

The activities of the majority of the *bZIP* family members in tomatoes were investigated by Zhu and coworkers, and basic region/leucine zipper (*bZIP*) transcription factors function as essential regulators in ABA-mediated stress response in plants [33]. *SlbZIP1* may have potential uses in the creation of salt- and drought-tolerant tomato cultivars, according to the researchers, who hypothesized that *SlbZIP1* performs a critical function in salt and drought stress tolerance through altering an ABA-mediated pathway.

The expression of *SlMYB102* was shown to be higher in ripe fruits and roots of tomato plants than in vegetative organs by Wang et al. [58]. Authors discovered additional regulatory components that are photo-responsive, abiotic stress-responsive, and hormone-responsive in the *SlMYB102* promoter region. They emphasized that *SlMYB102* may be implicated in the pathways for proline synthesis and C-repeat binding transcription factor (CBF), which boost tomato plant cold resistance. The genomics to combat abiotic stress in tomatoes is given in Table 1.

## 5. Proteomics of Abiotic Stress in Tomato

Proteins regulate significant roles in plant stress regulation as cellular components, as well as in plant gene sequence, chemical metabolites, and regulation of nucleic acids or histones [10]. The evaluation of the complete expressed array of proteins in an organism over time and space is known as proteomics. Proteomics analyses provide a plethora of details about the expressed proteins in the form of proteome profiles that fluctuate with developmental stage, dietary adequacy, age, environmental circumstances, or organs [77]. Moreover, it helps in the identification and assessment of various stress (salinity, water, and temperature)-susceptible proteins [24,32], thereby comprehending their involvement in abiotic stress-induced signaling.

Plant stress tolerance was often measured using only the whole proteome; however, several proteome-related studies, including the organellar proteome, phosphorproteome, nuclear proteome, cell wall proteome, and proteo-genome, began subsequently [77,78,79]. Consequently, proteome profiling is performed in various ways, comprising MS (mass spectrometry, which is concerned with the evaluation of charge and mass of protein fragments, resulting in the determination of any modifications in the peptide sequence), MALDI-TOF, or two-dimensional gel electrophoresis (2-DGE) [32]. Zhou et al. [80] determined the application of proteomics in tomatoes subjected to long-duration of stress conditions, further integrated with aluminum proteomes. Moreover, it revealed the modifications in the root proteins, thereby affecting mineral uptake mechanisms, physiological processes, and root development strategies. Furthermore, protein function is determined by protein biological interactions such as protein cellular localization (descriptive proteomics), post-translational and transcriptional (PTMs) changes, and protein–nonprotein relationships [13,81]. Even though several proteome analyses have been conducted to explore abiotic stress tolerance in tomatoes, data evaluation remains a roadblock on the route to in-depth proteomics research in crops.

Although proteomics is an effective way to study how organisms respond to constraints, it still has inherent flaws in aspects of reproducibility, the heterogeneity of the compounds being analyzed in contexts of abundance range, and physicochemical characteristics [15]. When scientists use species whose genomes have not yet been completely sequenced, the identification of proteins might potentially present problems. It is also still a time-consuming process. However, better protein descriptions are becoming accessible in public databases. The latest proteomics advances will aid in the identification of additional regulatory target proteins, leading to the creation of stress-tolerant crops with improved yield and quality [13].

### 5.1. The Fruit Proteome of Tomato

Since the beginning of human existence, the *Solanaceae* family of higher plants has been essential to human nourishment. It is one of the most commercially significant plant groups. Studies on proteomics have made major contributions to our understanding of the structure and function of *Solanaceae* species. Due to their economic significance, the majority of studies on Solanaceae have been concentrated on tomato, potato, and tobacco. Although it is highly challenging to extract stable protein combinations from plant tissues (due to an elevated quantity of proteases and other enzymes; secondly, secondary compounds generated in plant cells frequently obstruct further protein separation and downstream analysis [82]), fruit proteome is mostly studied in case of tomatoes. The significant determinant of fruit quality is ripening. To make it easier, the ripening process is categorized into several growing phases, viz. bred-red, light-red, turner, breaker, and green [81,83]. These are further investigated for ripening-associated proteins, modulated proteins, and cultivar-determined proteins. The evaluation process follows a series of low- and high-throughput processes beginning with a gel-based approach (2DE) that separates total fruit proteins of all the stages and detects a few spots that are differentially modified for analyzed stages. Additionally, quantitative reverse transcription-PCR (qRT-PCR) analyses and microarray-based transcriptomics were combined with proteomic data to identify additional genes and proteins, an ethylene-responsive transcriptional coactivator, glutathione S-transferase (GST)/peroxidase, oxalyl-CoA decarboxylase, etc. [84].

A cherry tomato pericarp proteome study revealed an increase in proteins involved in protein formation or amino acid breakdown during cell division. During the cell growth stage, it was discovered that proteins involved with photosynthesis and cytoskeleton synthesis were momentarily expressed, but proteins involved in glucose metabolism and oxidative reactions were elevated during fruit growth [85].

### 5.2. Protein Cellular Localization (PCL)

Because they are directly involved in generating novel phenotypes by altering physiological characteristics to altered environments, proteins play a significant role in plants’ stress responses. Almost every organelle in plants has a specific function during stress adaptation. The location of proteins within cells is tightly controlled and essential to their function. For instance, the nucleus is where stress signals are translated into gene expression, and the mitochondria and chloroplast are the most metabolically active sites that are responsible for the supply of energy. The four main cell fractions—nuclear, microsomal, mitochondrial, and plastidial—are separated using traditional methods of cellular separation using differential centrifugation [13]. Disease resistance, persulfidation, photosynthesis, defense mechanisms, metabolism, stress, and protein production are all activities in which these proteins are engaged [35,86,87].

To fully understand the localization patterns of all proteins that are involved in a particular cell type, the technique of spatial proteomics is used. Among these, the imaging-based approach is the most capable to provide a direct view of the in situ localization of each protein. It necessitates a complete collection of cell lines producing tagged proteins, a high-performance microscope setup, and a proteome-wide collection of binding reagents [88,89]. Protein binding partners are discovered by coimmunoprecipitation [90] or the increasingly common proximity ligation by APEX or BioID [91,92], in conjunction with mass spectrometry, for spatial proteomics through interaction networks. Mass spectrometry quantifies the dispersion of proteins among the fractions; cluster analysis reveals that proteins linked to identical organelles have comparable abundance distribution characteristics. This method is ideal for the determination of protein translocations since it does not call for any special tools or cell types, is relatively quick and easy to use, and is completely noninvasive [13].

Organellar mapping using proteome profiling is focused on the fractionation of lysates to obtain partially separate organelles and emerges among several strategies as being the one capable of identifying the cellular localizations of numerous proteins in a single trial. Significantly, it can also track protein transport between cellular components, offering a dependable systems investigation tool for analyzing both healthy and unhealthy biological processes [93]. Another method, i.e., employing diverse baits to distinguish specific compartments, results in extremely thorough organellar inventories that eventually link to generate a complete cell map.

### 5.3. Role of Protein Isoforms and Post-Translational Modifications (PTMs)

PTMs have a crucial role in controlling protein stability, activity, and cellular localization. Significant alterations in the PTMs of certain proteins are always linked to plants’ tolerance to abiotic stress [94]. Moreover, 20 distinctive amino acid arrangements—the post-translational modifications produced by incorporating different chemical elements such as carbohydrates, phosphates, lipids, and even other proteins—are the root of the formation of protein structure. However, the formed series of proteins differs based on cell type, shape, function, residing tissue, surroundings, and most importantly the developmental stage of the cell. Furthermore, proteins have variable isoforms that are metabolized, degraded, spliced, and frequently incorporated with one another to create certain complexes. The proteome profiles of two tomato cultivars (regional and commercial elite ecotype) were examined by Rocco et al. [83] as they ripened. The two cultivars share redox, stress, and energy-producing proteins such as various GST isoforms, thioredoxin peroxidase 1, HSPs, perhaps adenylate kinase 2, malate dehydrogenase cytosolic, meta caspase 1, etc.

Most responses include several different protein isoforms. Protein modifications in succeeding biochemical processes typically need close coordination, such as in the biosynthesis of isoprenoids and carotenoids. For instance, fruit peel and meat express isoforms such as GGPS1 and GGPS2 or IPI1 and IPI2 in quite different ways [34].

Plant molecular responses to abiotic stress are frequently seen as a complicated process that is mostly centered on modifying the transcriptional activity of stress-related genes. Although hundreds of PTMs have been recognized, it is difficult to analyze all potential protein alterations. Plant phenotyping for crop improvement monitors and reports plant responses to abiotic stress at the PTM level [94]. Moreover, it is capable of providing a better ability to comprehend the procedures of agricultural plant stress acclimation and stress tolerance acquisition as a result of the large-scale identification and quantification of PTMs.

### 5.4. Proteome Description of Protein Biological Function

As mentioned earlier, a proteome is the blanket term that refers to the collection of proteins expressed by the genome including post-translational modification. It is neither constant nor homogenous. Protein identification, quantification, localization, PTMs, and functional, structural, and protein–protein interactions are often the prime focus of any proteomic investigation. However, proteomics not only reveals information about the intricacy of life, but it also offers importance to the vitality of cells and their response to varying topographical or climatic patterns. While many of the PTMs have been investigated at the transcript level, quantifying protein abundance, which is closely connected to enzymatic activity, might give a more reliable indicator of protein function [34].

Proteomic research also gives a global perspective of the mechanisms underpinning healthy and pathological cellular functions [95]. Despite decades of physiological and molecular investigation of the biological system, systems biology techniques will keep revealing links between cell metabolism and processes that were neither obvious nor anticipated [96]. The various proteins and their functions in the stress tolerance of tomato are given in Table 2.

## 6. Metabolomics of Tomato in Stress Management

### 6.1. Metabolomics Technologies and Advancements

With the advent of genomics, transcriptomics, and proteomics, we have been able to understand a lot about various plant diseases and the mechanisms underlying their causes. However, metabolomics enables the assessment of biological processes under complex environments. The total number of metabolites in the entire plant kingdom ranging from polar to nonpolar, volatile to nonvolatile is estimated to exceed 200,000. To analyze all of a metabolite’s chemical features due to its extreme diversity, one must integrate various analytical systems [112]. The various metabolites responsible for stress tolerance in tomato is given in Table 3.

Applications of separation-based methods such as liquid/gas chromatography (LC/GC) paired with mass spectrometry (MS) and/or in conjunction with nuclear magnetic resonance (NMR) and Fourier transform ion resonance (FTIR) have helped in the documentation and quantification of metabolites in both untargeted and targeted manner. In targeted metabolomics, a finite list of compounds is selected with reference standards for precision in analysis and unambiguous identification of metabolites, whereas untargeted metabolomics aims to profile a broad range of metabolites having unique features [113]. While targeted metabolomics is limited by the availability of reference standards, untargeted metabolomics is limited to the low rate of identification of metabolites with known structures, unsatisfying repeatability, and the requirement of complex data processing. As such, a semitargeted approach, also known as suspect screening analysis, provides a mid-way to scan for metabolites based on compound-specific information, without the use of reference standards [114,115]. Again, by merging the advantages of both targeted and untargeted metabolomic approaches, another method, known as pseudotargeted metabolomic has emerged. In this approach, ion pairs of metabolites for multireaction monitoring (MRM) using MM-Ion Pair Finder software for TQMS are automatically defined without the necessity for reference values [116].

Single-cell metabolomic studies have also gained momentum in recent years. Protoplasts of plant cells have been isolated for single-cell studies [117]. Recent trends in single-cell metabolomic studies involve direct sampling from plant cells in a minimally disruptive manner to the cell and its microenvironment. These include pipette-mediated aspiration to target cells [118] and probes with micrometer tips attached to electrospray ionization for penetrating cells and sucking out cell contents, such as picoliter pressure-probe electrospray-ionization mass spectrometry [119] and internal electrode capillary pressure-probe electrospray-ionization mass spectrometry [120].

By using mass spectrometry imaging (MSI), which does not need chemical or antibody labeling, it is possible to visualize the geographic distribution and relative abundance of molecules. The MSI techniques that are routinely utilized nowadays include laser desorption/ionization (LDI), desorption electrospray ionization (DESI), secondary ion mass spectrometry (SIMS), matrix-assisted laser desorption ionization (MALDI), and laser-ablation electrospray ionization (LAESI). The excellent spatial resolution of SIMS MSI is by far its greatest advantage over other methods. While the preparation of the sample, the shape and focus of the laser beam, the application of the matrix, and the quality of the matrix all play a role in achieving excellent spatial resolution in MALDI MSI, the usage of matrix-free MSI methods such as LDI, DESI, and LAESI is gaining popularity [121,122].

Metabolomics, in particular, is a useful method for evaluating stress responses across plant species since primary metabolite structures are universal and specialized metabolite structures are largely conserved. With the technological advancements in mass spectrometry and spectroscopy, highly complex data are being generated. Higher complexity in data may lead to a higher false discovery rate. As such, to analyze these complex big data, new software tools, packages, databases, and resources are constantly being developed [123]. Recent technology developments, deep learning (DL) and machine learning (ML)-based methodologies, and in-depth multiomics data analysis approaches are enabling researchers to construct detailed metabolic reports and models for the desired plant species under particular settings. To provide a roadmap for the future generation of crops that are resistant to environmental deterioration, such precise metabolic descriptions are necessary.

### 6.2. Metabolomics Applications in Plant Stress Responses

Plant stress is defined as any alteration in growth circumstances that disturbs metabolic equilibrium and necessitates the adaptation of metabolic pathways by acclimation. Metabolites are spatially distributed across various tissues, organs, and cellular compartments. Plants are regularly disputed by diverse environmental factors that comprise abiotic and biotic stimuli, which as a response cause an alternation in the composition of their metabolites. These obstruct the overall growth and development of plants and productivity, which subsequently intimidates food security, specifically while considering the burden of the increase in the worldwide population. Metabolomics can render insight into plant metabolism during the developmental procedure and in response to lots of stresses by identifying various substances, such as derivatives of stress metabolism, stress signal transduction molecules, or molecules belonging to the acclimating response of plants; it can also aid in the guided explanation of stress biology in plants.

While primary metabolites are essential for plant growth and development and are well conserved in their molecular structures and abundances across the plant kingdom, secondary metabolites and their regulation are vulnerable to environmental fluctuations, including extremes of light, temperature, and water, salinity, UV, nutritional shortages, heavy metals, and oxidative stress [124,125,126], as well as contact with other plant species [127]. These strains change how the genes responsible for their synthesis express themselves [128,129]. Plants under stress tend to synthesize several secondary metabolites to cope with their adverse effects by permitting metabolic adjustments and restoring plant homeostasis [130]. It is therefore imperative to understand the dynamics of plant secondary metabolites in response to stress.

### 6.3. Plant Metabolites and Ecological Adaptation

Plants synthesize a large repertoire of metabolites. Besides the production of primary metabolites necessary for growth, plants also produce a diverse assortment of secondary metabolites. Being sessile and devoid of an immune system, a plant’s survival and reproductive fitness solely rely on its ability to adapt. In the course of evolution, ecological challenges have led plants to develop survival strategies in the guise of secondary metabolites. To ward off herbivorous insects and vertebrates, plants belonging to the *Solanaceae* family have evolved secondary metabolites such as tropane alkaloids, steroid alkaloids, nicotine, and withanolides [131]. The genus *Capsicum* of the *Solanaceae* family evolved lineage-specific biosynthesis of capsaicinoids to deter herbivory [132]. The evolutionary aspect of the secondary metabolites is further reinforced by the positive correlation between geographical distribution and the presence of calystegines in some genera exclusive to the Solanaceae family that have South America as their center of diversity [133]. Calystegines are secondary metabolites resulting from the tropane alkaloid biosynthetic pathway [134] that influence rhizosphere ecology as nutritional sources for soil microorganisms [135]. It has been widely recognized that rhizosphere microorganisms promote plant growth and protect plants from abiotic and biotic stresses [136]. Acyl sugars are another plant-protective insecticidal secondary metabolite produced in the Solanaceae family. The broad set of evolutionary mechanisms such as gene duplication and neofunctionalization of these enzymes has led to their structural diversification both within and beyond tomato [137]. The glycoalkaloid saponin α-tomatine is a tomato-specific secondary metabolite that stimulates programmed cell death facilitated by reactive oxygen species [138] and is found abundantly in green tomatoes, but is reduced as the fruit matures [139]. *Nectria haematococca*, a fungus that colonizes red tomatoes but not the green ripe ones, expresses the 2-tomatinase gene present in *Septoria lycopersici* and *Colletotrichum coccodes*, allowing it to detoxify tomatine and parasitize green tomatoes [140].

### 6.4. Plant Metabolites in Response to Stresses

#### 6.4.1. Metabolomics of Plant–Microbe Interactions

Tomato plants have served as model systems for the investigation of several plant–microbe interactions. The interactions between plants and microbes can either be beneficial or pathogenic. Plant growth-promoting rhizobacteria such as *Pseudomonas fluorescens* N04 and *Paenibacillus alvei* T22 induce disease resistance of tomato plants towards *Phytophthora capsici* through tissue-specific metabolic changes in phenylpropanoids, benzoic acids, glycoalkaloids, flavonoids, amino acids, organic acids, and oxygenated fatty acids [141]. Treatment of tomato seeds with *Trichoderma harzianum* released secondary metabolites of 6-pentyl-2*H*-pyran-2-one, and harzianic acid enhanced acetylcholine as well as γ-Aminobutyric acid (GABA) content and stimulated seed germination rate and seedling growth) [142]. Moreover, the treatment of tomato plants with the natural resistance inducer hexanoic acid primed the pathogen resistance of tomato plants towards *Botrytis cinerea* and *Pseudomonas syringae* by bringing perturbations in amino acids, sugars, and free fatty acids, along with primary and secondary metabolism [143]. The bacterium *Bacillus fortis* induces system resistance against *Fusarium* wilt by phenylacetic acid-mediated remodulation of tomato metabolic networks along with defense-related pathways and upregulation of various phenylpropanoid precursors [144].

Phenolics are the most pronounced secondary metabolites found in plants, which are produced mainly via the shikimic acid and malonic acid pathways through phenylpropanoid metabolism. Phenols are broadly classified into flavonoids and nonflavonoids [136]. These include flavones, isoflavones, anthocyanins, tannins, lignin, salicylic acid, furanocoumarins, etc. [145,146]. The phytopathogen *Ralstonia solanacearum* causes lethal wilt disease in tomatoes. It enters the plant through the roots, invades the xylem vessels, and rapidly colonizes the entire stem. Upon reaching a threshold of bacterial density within the plant, the bacterium disrupts plant physiology and induces plant death. Quantitative dynamics of xylem content during colonization revealed glutamine and asparagine as the preferred choice substrates of *R. solanacearum* [147]. Metabolomic profiling of tomatoes via untargeted metabolomics indicated the importance of the phenylpropanoid pathway in tomato defense response to *R. solanacearum* infections. An increase in fold changes across different tomato cultivars was observed with amino acid levels in the leaves, flavonoids in the roots, hydroxycinnamic and organic acids in the stems, and hydroxybenzoic acids and phytoalexins throughout the tissue metabolomes [148]. The accumulation of the phenyl amide phytoalexin putrescine was in line with the observations made by Gerlin et al. [147]. The alteration in the volatile organic compound (VOC) spectra of tomato plants by *Candidatus Liberibacter solanacearum* influenced the settlement behavior of Psyllids, a bacterial vector [149]. According to metabolomics based on 1H nuclear magnetic resonance (NMR), when the tomato is locally infected with the tomato mosaic virus, its quantities of amino acids and sucrose as well as phenolic acids decrease while its contents of tricarboxylic acid increase. Conversely, systemic reactions showed comparable metabolic profiles but higher levels of tryptophan, sucrose, and caffeoyl esters of glucaric acid [150]. The cleavage of α-tomatine by *Fusarium oxysporum* CfTom1 glycosyl hydrolase resulted in the accumulation of tomatidine and tetra-saccharidelycotetraose that suppressed induced defense responses in tomato plants [151]. This was further backed by the demonstration of lower virulence in tomato plants by the tomato leaf mold *Cladosporium fulvum* with suppressed CfTom1 activity [152].

#### 6.4.2. Metabolomics of Plant–Herbivore Interactions

Plants are a nutritious source of food, and are thus consumed by organisms that are anatomically and physiologically adapted to eat them. To fend off these phytophagous organisms, plants synthesize an extensive range of metabolites, either constitutively or in response to herbivore attacks. These responses triggered in plants can again be either localized or systemic. Finding plant secondary metabolites that provide resistance against herbivores is probably where metabolic investigations of plant–herbivore interactions are most frequently used.

Tomato plants are very prone to *Tuta absoluta* and respond through the massive, localized accumulation of phenol amides and by upregulating putrescine hydroxycinnamoyl transferases upon herbivory by its larvae [153]. A study amongst tolerant and susceptible tomato plants exposed to *T. absoluta* infection revealed the accumulation of several organic acids in the tolerant plants [154]. According to this theory, a comparison of *T*. *absoluta*’s differential reactions in tomato plants and eggplant found considerably lower levels of total phenols, jasmonic acid, fructose, and sucrose as well as amino acids, fructose, and sucrose in tomato than in eggplant [155]. Colonization of tomato root by mutualistic microbes *Trichoderma harzianum* and *Rhizophagus irregularis* brought changes to the shoot metabolome by the enhanced accumulation of secondary metabolites such as steroidal glycoalkaloids and altered patterns of fatty acid amides and carnitine-derived metabolites, thereby impairing the development of the insect herbivore *Manduca sexta* [156]. The secondary metabolite tannin impedes the digestion of plant materials by binding to salivary proteins and digestive enzymes of insects by blocking or interfering with protein activity. A negative correlation between the concentration of tannins in tomato seedlings and the number of *Trialeurodes vaporariorum* was reported by Bialczyk et al. [157]. Lignin is a highly branched heterogenous polyphenol found in the secondary cell wall of plants. It acts as a physical barrier toward the chemical and biological degradation of cell wall polysaccharides due to its inherent rigidness and insolubility. Furanocoumarins are another set of phenolic compounds that can be highly toxic to certain herbivores due to their integration into the DNA, which leads to rapid cell death [145].

The phytohormone jasmonic acid imparts resistance to tomato plants against a wide range of herbivorous attacks, including *Spodoptera littoralis*, *Tetranychus urticae*, and *M. sexta* larvae [158,159,160]. The signaling peptide systemin starts a signaling cascade upon injury that results in the production of jasmonic acid from linolenic acid [161]. When leaves are developing normally, plants produce and store a variety of volatile terpenoid chemicals in specialized storage organs called glandular trichomes [162]. Herbivore wounds frequently cause the production of these terpenoids, which trigger plant defenses either directly or indirectly [163]. Herbivory also stimulates the formation of volatile esters such as methyl salicylate, which is made through the shikimic acid pathway, and green-leaf volatiles (GLV), which are made from linoleic or linolenic acid through the use of lipoxygenase and hydroperoxide lyases. Jasmonic acid and systemin are once again implicated in the emissions of these volatile organic compounds (VOCs), according to several investigations [158,159]. To protect tomato plants against *Bemisia tabaci,* salicylic acid, an immediate precursor of methyl salicylate, was administered exogenously. This boosted the levels of methyl salicylate and -limonene [164]. *B. tabaci* serves as an insect vector for the TYLCV virus (Tomato yellow leaf curl virus) that causes tomato yellow leaf curl disease (TYLCD). High flavonoid levels in tomato plants were found to impact *B. tabaci* oviposition, settling, probing, and phloem-feeding efficiency, thereby reducing TYLCV spread [165].

However, herbivory is not limited only to the aerial parts. Several phytophagous nematodes feed on the root of plants, including tomatoes. Through altered root exudation resulting from the mycorrhizal association of *Glomus mosseae* and tomato, the penetration of the root-knot nematode *Meloidogyne incognita* was significantly reduced [166]. Intercropping Crown daisy (*Chrysanthemum coronarium*) with tomato reduces *M. incognita* infestation due to the exudation of lauric acid by crown daisy roots [167].

Besides secondary metabolites and VOCs, increasing evidence suggests that to tolerate herbivory, plants induce vast changes in their primary metabolism and reallocate new and existing resources to storage tissues. By mimicking herbivory, Gomez et al. [168] demonstrated how the application of methyl jasmonate, a known defense elicitor, to tomato leaves increased the sink of carbon and nitrogen from treated leaves to the roots. Similar findings by Gomez et al. [169] were also made upon the application of *M. sexta* caterpillar regurgitation as a mimic of herbivory. Here, they draw attention to the fact that, despite an increase in the root sink strength, the concentrations of metabolites tended to drop in the roots. This is presumably because soluble sugars were rapidly converted into starch pools for quick use to support root respiration by increasing nutrient uptake. Contrastingly, the concentration of metabolites tended to increase in the apex. In line with these findings, Steinbrenner et al. [170] demonstrated how changes in the metabolome of tomato plants are highly tissue-specific and herbivore-specific when larvae of *M. sexta* and *Helicover pazea* are allowed to feed on it.

#### 6.4.3. Impact of Pesticides and Other Chemicals and Epigenetic Modifications on the Metabolomics of Tomato

Treatment of plants with plant hormones and pesticides to strengthen their defense, overcome stress, and increase their quality and yield is a widespread conventional agricultural practice. There are few publications, nevertheless, on how these chemical agent treatments affect plant metabolites. Reduced rates of photosynthesis and stomatal conductance, impaired glucose turnover, and changed cell wall composition in tomatoes are the outcomes of exogenous administration of Ethephon (2-chloroethyl phosphonic acid, or CEPA), an ethylene-releasing substance, to promote fruit harvesting [171]. By increasing the activity of antioxidant enzymes such as superoxide dismutase, catalase, ascorbate peroxidase, and glutathione reductase, as well as proline and glycine betaine osmolyte production and flavonoid accumulation, jasmonic acid and nitric oxide supplementation help reduce salt stress [172]. By modifying the tomatoes’ osmoregulatory systems, polyamine metabolism, organic acid secretion, chlorophyll metabolism, antioxidant levels, and photosynthetic efficiency, exogenous foliar sprays of the polyamine spermidine help the plant survive low-iron stress. Several transcription factors (GH3, SAUR, ARF) that are connected to the growth hormone response in leaves and ethylene-related signaling factors (ERF1, ERF2) that are connected to roots are also upregulated under low-iron stress by spermidine application [173]. Chemical control is currently the most effective and reliable method in use for pest management. However, its excessive use also has adverse effects on plants. Shakir et al. [174] reported the induced oxidative stress in tomato seedlings associated with the excessive use of the pesticide emamectin benzoate, alpha-cypermethrin, and imidacloprid. In another study, elevated ROS levels due to pesticide overuse in tomatoes had reduced germination rate, root biomass, chlorophyll-a, chlorophyll-b, total chlorophyll, and carotenoid levels [175]. Furthermore, due to the environmental risks imposed by the rampant use of pesticides, several adaptive strategies to improve plant defense capacity by stimulating induced mechanisms have come to light. Triggering broad-spectrum defense priming in tomatoes by β-aminobutyric acid induced a durable resistance in tomato fruit against *Botrytis cinerea*, *Phytophthora infestans*, and *Pseudomonas syringae* [176]. Another approach is to use fluopimomide In conjunction with *Bacillus methylotrophicus* for disease management in tomatoes [177]. Fluopimomide is a succinate dehydrogenase-inhibiting fungicide with nematicidal activity against *Meloidogyne incognita*, which also promotes plant growth [178,179]. Sharaf and Farrag [180] demonstrated the use of low concentrations of indole-3-acetic acid (IAA) for attaining induced resistance against *Fusarium oxysporum lycopersici* due to improved plant growth. Natural pesticides such as azadirachtin have also attracted increased usage in organic agriculture in recent years. Recent findings suggest that it stimulates induced systemic resistance (ISR) in tomato plants very similar to *B. subtilis*-induced ISR [181].

The developmental trigger of fruit ripening in tomatoes is in itself an epigenetic switch [182]. The interplay among various epigenetic modifications regulates gene expression in the developmental processes of a plant. Genomic DNA methylation is an epigenetic process that alters gene expression via histone modification and chromatin remodeling [183]. Yang et al. [184] demonstrated how sequential DNA methylation and demethylation are indispensable for the normal production of tomato fruits. The DNA methyltransferase 1 (*SlMET1*) controls the expression of many development-related genes such as the ripening-inhibitor (*rin*) target genes, ensuring the normal production of flowers and fruits. The tomato *rin* mutant has lower levels of ethylene, organic acids, sugars, carotenoids, and amino acids [185]. The ethylene and carotenoid content of the tomato decreased because of spontaneous epimutations caused by methylation of the SQUAMOSA promoter binding protein-like (*SBP-box*) gene located at the colorless nonripening (cnr) locus, which prevented the tomato from ripening normally [186]. Once more, the Vitamin E content of ripe tomatoes is impacted by DNA methylation of a SINE retrotransposon in the promoter region of the Vitamin E 3 (*VTE3*) gene, which codes for 2-methyl-6-phytylquinol methyltransferase [187]. By either suppressing DNA methyl transferases or increasing demethylases, DNA demethylation, on the other hand, promotes tomato fruit ripening [186]. Demethylation and activation of genes necessary for fruit ripening, cell wall breakdown, production of fruit color and flavor chemicals, and ethylene biosynthesis and signaling depend on the tomato Demeter-like dnademeterase (*dml*) gene (*SlDML2*) [188].

Postharvest chilling of tomatoes to temperatures as low as 4 °C leads to the depletion of important flavor volatiles and reduced flavor quality due to transient cytosine methylations in ripening-related genes such as ripening inhibitor (*rin*), colorless nonripening (*cnr*), and nonripening (*nor*) [189].

Acetylation, phosphorylation, methylation, ADP-ribosylation, ubiquitination, sumoylation, and other processes can alter the amino terminus of histones [190]. Numerous studies on histone alterations claim that they play a part in either orchestrating or suppressing the ripening of tomato fruit. Among these modifications, histone methylation and acetylation are the two most characterized post-translational modifications in tomatoes. The *SlJMJ7* histone *H3K4* demethylase represses key ripening-related genes and the DNA demethylation (*DML2*) gene via H3K4me3 demethylation in tomatoes and inhibits ripening [191]. Conversely, the histone demethylase SlJMJ6 reportedly promotes tomato ripening by removing *H3K27* methylation [192]. The trimethylation of H3K27 was facilitated by the polycomb repressive complex 2 (*PRC2s*) that inhibited the initiation of ripening [193,194]. Two genes encoding the histone methyl transferase (HMT) enhancer of *zeste E(z)* in tomatoes, *SlEZ1* and *SlEZ2*, have components of the PRC2 complexes. The *SlEZ1* gene is silenced by RNA interference (*RNAi*), although this does not affect fruit or vegetative growth other than an increase in the number of carpels and aberrant stamen formation. On the other hand, throughout the growth and ripening of the fruit, the *SlEZ2 RNAi* plants exhibit changes in carpel initiation and fruit cuticle formation [195,196]. Moreover, nonhistone lysine methylation modification regulates tomato fruit ripening [197].

Reduced potassium dependency-3/histone deacetylase-1 (*RPD3/HDA1*), histone deacetylase-2 (*HD2*), and silent information regulator-2 (SIR2) are the three types of histone deacetylases (HDACs) found in plants [198]. RNAi-mediated silencing of the RPD3/HDA1 family histone deacetylase gene *SlHDA3* negatively regulates fruit ripening and carotenoid accumulation [199]. Zhao et al. [200] demonstrated tomato reproductive development associated with the interaction of SlHDA1, SlHDA3, and SlHDA4 with two MADS-box proteins TAG1 (TOMATO AGAMOUS1) and TM29 (TOMATO MADS BOX29). Besides ripening, silencing SlHDA5 also decreased seedling tolerance to salt, drought, and abscisic acid [201]. The HD2 family genes *SlHDT3* and *SlHDT1* also regulate tomato fruit ripening by affecting carotenoid accumulation and ethylene biosynthesis [202,203]. Histone acetyltransferase (HATs) encodes the *SlHAF1* gene, which is crucial for tomato fruit ripening [204].

The epigenetic modification of mRNA via N^6^-methyladenosine (m^6^A) plays multiple physiological functions in the development of plants. m^6^A controls nearly every aspect of RNA metabolism, including the stability of mRNA, including splicing, nuclear retention, mRNA export, translation efficiency, and 3′-end processing [205,206]. In response to cold stress, m^6^A induces pollen abortion through the deposition of higher levels of abscisic acid and the reduction in m^6^A levels in anthers, disruption in tapetum development, and pollen exine formation [207]. Hu et al. [208] demonstrated that an increase in global m^6^A levels led to the modification of a large number of fruit-expansion-related genes involved in hormone responses and endoreduplication during the expansion of tomato fruit from immature green to mature green. Conversely, direct injection of m^6^A writer and eraser inhibitors, viz. 3-deazaneplanocin A or meclofenamic acid, respectively, into tomato fruits altered m^6^A levels and suppressed tomato fruit expansion [208]. Besides being involved in reproductive development and fruit expansion, m^6^A also mediates tomato fruit ripening. The *cnr* ripening-deficient epimutant, which harbors hypermethylated DNA, also exhibits a global increase in m^6^A methylation and a reduction in expression of the RNA demethylase gene *SlALKBH2*. DNA methylation regulates *SlALKBH2*, which in turn binds to *SlDML2*. Mutation of *SlALKBH2* decreases the abundance of mRNAs of *SlDML2* and delays fruit ripening [209].

**Table 3:** Metabolites involved in abiotic stress tolerance in tomato.

Metabolites	Function	Result	References
Melatonin	Prevents damage to proteins and membranes	Tolerance against abiotic stress	[210]
ABP19a protein (drought-responsive auxin-binding protein (ABPs) family	Involved in many development processes and drought response	Drought tolerance	[211,212]
SpUSP, an annexin-interacting universal stress protein	Reduction in oxidative stress by preventing the generation of ROS, activation of several stress-responsive genes that causes the accumulation of some osmoprotective solutes	Drought tolerance	[213]
Solyc04g014600 (Universal stress protein)	Protein profiling of phloem and its exudates	Drought tolerance	[214]
SlSRN1 (*Solanum lycopersicum* stress-related NAC1)	Disease resistance response as well as resistance to drought and oxidative stress	Tolerance against abiotic stress	[215]
SlNAC4	Fruit ripening and carotenoid accumulation	Oxidative stress response	[216]
SlNAM2	Flower-boundary morphogenesis	Inhibits extreme water loss	[217]
SlNAC1	Increasing viral replication and chill tolerance through interactions with replication accessory proteins	Tolerance to lower temperatures and phytopathogens	[218,219]
GOBLET	Identifying the borders of compound leaves’ leaflets	Inhibits extreme transpiration	[220]
SlMAPK3	Enhanced germination rate as well as seedling growth; moreover, formed transgenic plants resulted in an improved chlorophyll content and root biomass accumulation under Cd2+ stress	Tolerance to cadmium stress along with drought tolerance	[65]
C2H2-Type Zinc Finger Protein	Overall growth as well as the development of plant tissues	Abiotic stress responses	[221]

## 7. Conclusions and Future Recommendations

Tomato is grown worldwide and ranks third in the globe behind potato and sweet potato and first in the list of canned vegetables. It is cultivated for its edible fruits, which may be eaten fresh or processed. It is a good source of vitamins A, B, and C, as well as minerals, and the antioxidant lycopene. Although there is a high demand for tomatoes globally, constraints such as biotic and abiotic factors alone or in combination are responsible for the low yield. To overcome these limitations, the techniques associated with omics are the best alternatives. The growth and productivity of crops are commonly affected by different abiotic stresses. Different omics-based approaches such as transcriptomics, proteomics, metabolomics, etc. have been successfully used either individually or in combination to understand the responses of plants against stresses. On commencement of the stress conditions, the plants accommodate themselves by changing the metabolic cascades and genetic regulations, ultimately leading to the expression of new genes. Hence, it is essential to elucidate the gene and its function to reveal the response of the plant toward stress. This can only be possible due to developed biotechnology and omics tools and techniques. Tomato is one of the most important crops globally. Numerous omics studies and integrated management strategies are presented in this review, and they provide information on the present situation and potential future directions for efficient control of abiotic stress in tomatoes. In a second Green Revolution, which may meet food demands and guarantee nutritional security for expanding populations, we forecast that the adoption of the above-mentioned technologies in tomatoes would transform agriculture and can offer better crop production to meet the need for food. We must take advantage of this chance to boost tomato production to fulfill the need for vegetables, and nutritional security globally, particularly in emerging nations.

## Figures and Tables

**Table 1:** Genomics to combat abiotic stress tolerance in tomato.

S. No.	Gene/Origin	Function	Expression/Regulation	Results	References
1	*SDG34*	Response to stress	Expression of negative stress response regulators and transcriptional repressors	Improvement of stress and pathogen tolerance	[51]
2	*SlGRAS10*	Increasing osmotic potential, flavonoid production, and the ROS scavenging mechanism to increase abiotic stress tolerance	Downregulation		[48]
3	*BEL1*-like genes	Numerous biological processes in plants are regulated by transcription factors, which are members of the superfamily of three-amino-loop-extension (TALE) proteins	Displayed various tissue-specific expression patterns and reacted to heat, cold, and drought stress	Plant growth and abiotic stress response	[59]
4	*SlAIM1*	Salt and oxidative stress tolerance	Salt and oxidative stress tolerance is increased by SlAIM1 overexpression, but these two abiotic stimuli are made more sensitive by SlAIM1 silencing	Resistance to abiotic stress	[52,60]
5	TFs, s Cycling Dof Factor *AtCDF3*, *AtDREB1a*, *NAC* transcription factor JUNGBRUNNEN1 (*AtJUB1*) and *AP2/ERF*-like transcription factor CcHRD	Increases abiotic stress tolerance of tomatoes, including cold, salt, and drought stress	Overexpression	Stress tolerance	[52,61]
6	*SlMBP8*, *SlHB2*, *SlAGO4A*	Tolerance to salt, drought stress	Overexpression	Tolerance to salt, drought stress	[62]
7	*INVINH1*	Tolerance to cold stress		Tolerance to cold stress	[63]
8	*SlMBP8*	more tolerance to drought and salt stress	Gene silencing	More tolerance to drought and salt stress	[62]
9	*SI PL*	Resistance to pathogenic *Botrytis cinerea* and prolonged shelf life		Resistance to pathogenic *Botrytis cinerea*	[64]
10	*SlbZIP1*	Salt and drought stress tolerance	Expression	Salt and drought stress tolerance	[3]
11	*SlMAPK3*, *SlMPK7 i*	Resistance to chilling, cadmium, and drought stresses	Overexpression	Resistance to chilling, cadmium, and drought stresses	[65]
12	PpSnRK1α)	Accelerated metabolism of reactive oxygen species via upregulating antioxidase gene expression and antioxidant enzyme activity	Overexpression	Salt resistance	[66]
13	*SlBZR1D*	Salt tolerance and upregulated the expression of multiple stress-related genes	Overexpression and upregulation	Salt tolerance	[33]
14	*SlNL33*	Ascorbate accumulation	suppressed expression	Stress tolerance	[67]
15	*SlHY5*	Cold tolerance	Overexpression		[68]
16	*MdSWEET17*	Drought stress response and the regulation of fructose.	Expressed in tomatoes	Drought stress	[69]
17	*SiDHN*	*Saussurea involucrata* dehydrin gene overexpression	Overexpression	Cold and drought tolerance	[70]
16	*SlHSP17.7*	Controlling Calcium Signaling and Phosphatidylglycerol Metabolism	Overexpression	Cold tolerance	[71]
17	*SlABIG1*	Salt stress negative regulator gene	Knockout	Salt tolerance	[72]
18	*Solyc03g020030*	Proteinase inhibitor-II	Gene silencing	Thermotolerance	[73]
19	*SlDEAD23* and *SlDEAD35*	Abiotic and biotic stress responses	Overexpression	Enhanced tolerance to salt and cold	[74]
20	*SlGRAS10*	Improved the expression of superoxide dismutase, peroxidase, and catalase to lessen the impact of reactive oxygen species	Downregulation by RNA interference	Abiotic stress tolerance	[48]
21	*SlLBD40*	A negative regulator of drought tolerance, it was implicated in JA signaling.	CRISPR/Cas9 targeted mutagenesis (knockout)	Drought tolerance	[75]
22	*SlMAPK3* *i*	Removing ROS buildup and increasing the expression of genes associated with ethylene signaling	Over-expression	Salt stress tolerance	[76]

**Table 2:** Proteins to combat abiotic stress tolerance in tomato.

S. No.	Protein	Function	Result	References
1	Systemin peptide	Sodium exclusion, antioxidant activity, protease inhibitor	Lower palatability for herbivores and high salt stress tolerance	[97]
2	COR15 protein	Folds and adheres to the chloroplast membranes to support leaf cells during freezing	Cold tolerance	[98,99]
3	P5CS protein	Involved in proline biosynthesis and inducible upon salt shock in drought-resistance	Drought and salt tolerance	[100,101]
4	Transcription factor of the ERF (ethylene-responsive factor) family	Reduces cell injury and enhances tolerance against cold stress	Cold, heat, and flood tolerance	[102]
5	SpPKE1	Interact with an F-box protein associated with drought tolerance	Drought tolerance	[103]
6	LEA proteins	Prevents membrane leakage, membrane and protein stabilization	Water balance and ion sequestration maintenance	[104]
7	Dehydrins (DHNs)	Enhances tolerance to salinity and drought stress	Salinity and drought stress	[105]
8	2,3-butanediol	Drought and chilling response	reduced harmful effects of abiotic stresses	[106]
9	E42 and LA3120	Stimulates plant growth and reduces stresses	Better plant growth under water stress	[107]
10	RING zinc finger	Plant growth and reducing abiotic stresses	Reduced stresses on plant	[108]
11	Golden 2-Like	Plant development and reducing abiotic stress	Reduced drought stress tolerance by lowering SOD, peroxidase	[28]
12	Histidine kinase	Reduces abiotic stresses	Maintaining cellular Na^+^ homeostasis	[109]
13	*SlbZIP1*	Reduces ABA-mediated stress	Reduced salt and drought stress	[33]
14	MdVHA-B	Better tolerance to drought stress	Increased tolerance	[110]
15	LeHSP21.5	Improved tolerance to tunicamycin-ER stress inducer	Increased tolerance	[111]

## Data Availability

The data presented in this study are available in this manuscript.
